# Influence of Gender, Dispositional Optimism, and Coping Strategies on Appearance-Related Distress Among Swedish Adults With Cleft Lip and Palate

**DOI:** 10.1177/10556656211025196

**Published:** 2021-06-17

**Authors:** Anna Paganini, Martin Persson, Hans Mark

**Affiliations:** 1 Department of Plastic Surgery, Institute of Clinical Sciences, 70712 Sahlgrenska Academy, University of Gothenburg, Sweden; 2 Department of Reconstructive Plastic Surgery, 70712 Sahlgrenska University Hospital, Gothenburg, Sweden; 3 Department of Health and Society. Kristianstad University, Sweden

**Keywords:** cleft lip and palate, appearance-related distress, DAS24, LOT-Rs, Brief-COPE, optimism, coping

## Abstract

**Objective:**

To investigate the influence of gender, dispositional optimism, and coping strategies on appearance-related distress among individuals with unilateral cleft lip and palate (UCLP).

**Design:**

Cross-sectional design with self-report questionnaires analyzed primarily with Spearman correlations (*r*_s_) and multivariate regression analyses.

**Setting:**

A tertiary cleft center in Sweden.

**Participants:**

Eighty individuals with UCLP born 1966 to 1986. The mean age for men (n = 50) and women (n = 30) was 38.8 and 37.4 years, respectively.

**Main Outcome Measures:**

The Derriford Appearance Scale 24 measured appearance-related distress, the Life Orientation Test–Revised, short version measured dispositional optimism and pessimism, and the Coping Orientation to Problems Experienced, short version included 14 coping strategies.

**Results:**

Women had higher appearance-related distress than men, which was significantly (*P* < .05) related to self-blame (*r*_s_ = 0.59), pessimism (*r*_s_ = 0.59), and low optimism (*r*_s_ = −0.56). Men’s appearance-related distress was significantly associated with low active coping (*r*_s_ = 0.35), low use of emotional support (*r*_s_ = 0.29), denial (*r*_s_ = 0.39), behavioral disengagement (*r*_s_ = 0.41), and pessimism (*r*_s_ = 0.28). The only significant gender interaction reflected greater impact of optimism in reducing appearance-related distress for women (β = −0.06).

**Conclusions:**

This study showed that high levels of dispositional optimism decrease appearance-related distress, particularly for women. The coping strategies used differed between men and women, and the results suggest that both gender and psychosocial facto *r*_s_ need to be considered in regard to appearance-related distress among individuals with UCLP in both clinical and research settings. A possible way to decrease distress is to strengthen positive coping strategies and dispositional optimism.

## Introduction

Cleft lip and palate (CLP) is the most common congenital facial anomaly with a prevalence of approximately 1 in every 500 children born in Sweden (Hagberg et al., 1998). Being born with CLP affects both the appearance of the face and the functions of the palate, such as speech and occlusion. Treatment consists of reconstructive surgeries, as well as orthodontics and speech therapy, aiming for good functional results (Marcusson et al., 2002).

A cleft lip affects an individual’s facial appearance, and women with a cleft seem to be more dissatisfied with their appearance and request more revision surgeries than men (Marcusson et al., 2002; Mani et al., 2010; Mani et al., 2013; Paganini et al., 2018). A qualitative study of adults born with cleft reported that women are more concerned than men about appearance (Stock et al., 2016a). In the general population, the same phenomenon transpires; while both men and women experience poor body image, women are disproportionately more affected (Wang et al., 2019; Lacroix et al., 2020), which can be explained by cultural and societal norms (Strahan et al., 2006).

Previous studies among individuals with cleft have also reported gender differences in life satisfaction and psychological and appearance-related distress (Marcusson, 2001; Paganini et al., 2020). Women with cleft are more affected by psychological and appearance-related distress and have a lower degree of life satisfaction than men (Marcusson, 2001; Mani et al., 2010; Paganini et al., 2020). However, the mechanisms behind appearance-related distress among adults with cleft and the importance of gender in this regard are not yet fully understood.

In relation to individuals born with visible differences, including CLP, it is the subjective experience of the individual about the result of their surgery that predicts their psychological well-being, not the healthcare professionals’ opinion or any other objective measure (Semb et al., 2005; Sinko et al., 2005; Ong et al., 2007). It has also been previously shown that individuals with cleft who are dissatisfied with their facial appearance have lower health-related quality of life than those who report a higher satisfaction with their facial appearance (Marcusson et al., 2002). However, appearance-related distress among individuals with CLP is not associated with objective outcomes or previous treatment (Mani et al., 2010), thereby making subjective perception the most important factor. Thus, it is important to understand how perception is influenced by an individual’s personality and coping skills.

An individual’s personality consists of a multitude of personality traits, which describe enduring personality characteristics that affect an individual’s behavior in any given situation (Lazarus and Folkman, 1984). Dispositional optimism influences how a person reacts to stressful situations, and it is a psychological resource that is also associated with improved physical health and general well-being, as well as higher levels of perceived health and quality of life (Carver and Scheier, 2014; Scheier and Carver, 2018). Dispositional optimism is defined as the relative stable expectation that positive outcomes will occur across important life domains, and it is thought to remain fixed over time (Scheier and Carver, 1985; Scheier and Carver, 2018). It consists of 2 factors: optimism and pessimism that reflect confidence versus doubt regarding life in general (Muhonen and Torkelson, 2005; Herzberg et al., 2006; Carver and Connor-Smith, 2010). The exact relationship between optimism and pessimism is unclear, but they appear to be related to health in different ways, creating 2 different factors (Scheier and Carver, 2018). It seems that a high level of optimism and/or a low level of pessimism functions protectively for the individual, while a low level of optimism and/or a high level of pessimism is harmful (Serlachius et al., 2015; Scheier and Carver, 2018).

A multinational population-based study covering 142 countries found that the most optimistic individuals worldwide are young, female, and highly educated (Gallagher et al., 2013). However, in population studies from Germany and Norway, marginal to no gender differences in dispositional optimism have been found (Glaesmer et al., 2012; Hinz et al., 2017; Schou-Bredal et al., 2017). Regardless of gender, pessimism seems to increase with age (Hinz et al., 2017; Schou-Bredal et al., 2017). Examining dispositional optimism to assess psychological adjustment in the population with cleft is currently used in the United Kingdom as a core outcome measure (Stock et al., 2016b; Stock et al., 2020b).

Dispositional optimism is considered a relatively stable trait, but individuals also utilize more dynamic ways to react and handle a situation via different coping strategies (Carver and Connor-Smith, 2010). Coping is defined by Lazarus and Folkman (1984, p. 141) as “constantly changing cognitive and behavioral efforts to manage specific external and/or internal demands that are appraised as taxing or exceeding the resources of the person.” The dynamic character of coping ensures that an individual uses different ways of coping depending on the situation. The different strategies of coping encompass the approaches that individuals use in a stressful situation, including problem- and emotion-focused strategies (Lazarus and Folkman, 1984). Problem-focused strategies of coping are more commonly used when conditions are appraised to be controllable and amenable to change, while emotion-focused strategies aim to provide the means to handle a stressful situation that is deemed unchangeable (Lazarus and Folkman, 1984; Tamres et al., 2002). Examples of problem-focused strategies include active coping, planning, and seeking instrumental support, while emotion-focused strategies include, for example, denial, self-blame, seeking emotional support, and self-distraction (Lazarus and Folkman, 1984). Optimists and pessimists tend to differ in their choice of coping strategies, where an optimistic person uses problem-focused coping strategies more often than pessimistic persons (Scheier et al., 1994). In addition, gender differences can be found in the choice of coping strategies (Folkman and Lazarus, 1980; Tamres et al., 2002). Women are more prone to using emotional strategies that express their feelings to others (seeking emotional support) and the self (rumination, positive self-talk) (Folkman and Lazarus, 1980; Carver et al., 1989; Tamres et al., 2002; Matud, 2004). Overall, women report a greater use of most coping behaviors as well as using a greater variety of coping strategies than men (Tamres et al., 2002). When examining coping mechanisms in a general Swedish population, women were more prone to using strategies such as self-distraction, denial, emotional support, instrumental support, and venting than men (Muhonen and Torkelson, 2005).

Individuals with CLP live with a visible difference, the psychological impact of which creates a need for coping (Egan et al., 2011). In regard to living with cleft and the coping strategies utilized, previous studies have focused on coping strategies among parents of children with CLP (Stock et al., 2020a) and coping among children and adolescents living with cleft (Berger and Dalton, 2009; Berger and Dalton, 2011). In the context of adults with cleft, some qualitative studies illustrate the coping strategies used by the population (Egan et al., 2011; Stock et al., 2016a; Kappen et al., 2019). These studies mention positive coping strategies, such as having inner strength, focusing on one’s strengths, and having a positive outlook on life (Egan et al., 2011; Stock et al., 2016a; Kappen et al., 2019). However, negative coping strategies, such as avoidance and lack of social confidence, have also been described (Stock et al., 2016a; Kappen et al., 2019).

Living with a visible difference, such as CLP, may cause appearance-related distress in both men and women, regardless of the objective aesthetic result. There seems to be gender differences in appearance-related distress, where women report more distress than men. It is, therefore, of interest to investigate whether the subjective appearance-related distress can be explained by differences in personality and coping strategies among both men and women.

### Aim

The aim of this study was to investigate if appearance-related distress was influenced by gender, dispositional optimism, and coping strategies among adults with unilateral cleft lip and palate (UCLP).

## Methods

### Patients

A total of 59 women and 121 men born between 1966 and 1986 with UCLP and no associated syndromes or malformations were identified through the medical records at the cleft center of Sahlgrenska University Hospital. Two deceased male individuals, 7 with unknown addresses (5 males), and 9 emigrated (7 males) were excluded. The remaining 162 individuals received an information letter together with the study questionnaires and a prepaid return envelope. The individuals who did not respond were reminded by a phone call, and thereafter, 2 additional reminders were sent via post. The study was conducted from 2013 to 2014.

The cleft care at Sahlgrenska University Hospital is extended to all children born with cleft in west Sweden, from birth to 19 years of age, through a multidisciplinary team that has been in place since the mid-1960s. Between 1966 and 1975, the team consisted of a plastic surgeon and an orthodontist, and in 1975, a speech and language pathologist was added. The follow-up protocol is standardized with clinical evaluations from the different professionals at 3, 5, 7, 10, 13, 16, and 19 years of age. After 19 years, the former patients are always welcomed back for further evaluations and treatment. During the clinical evaluations, the need for further corrective surgery is evaluated and patients are offered surgical revisions to the lip and nose as well as orthognathic surgery as needed.

### Measures and Instruments

#### Life Orientation Test–Revised, short version

We used the revised Life Orientation Test, short version (LOT-Rs) to measure dispositional optimism and pessimism (Carver, 1997; Schou-Bredal et al., 2017). The LOT-Rs is a viable instrument for assessing people’s generalized sense of optimism and pessimism (Scheier et al., 1994). We used the Swedish version of LOT-Rs, translated by Muhonen and Torkelson (2005). The LOT-Rs is a 6-item measure of individual differences in optimism and pessimism. Three items are positively worded, and 3 are negatively worded. A sample item from the scale is, “I am always optimistic about my future.” The respondents are asked to rate the extent of their agreement with each item, using a 5-point Likert scale ranging from 0 to 4. Two subscales are created, each using the positively and negatively worded items, creating separate scores for optimism and pessimism (Carver, 1997; Muhonen and Torkelson, 2005). Each subscale ranges from 0 to 12 points. The Swedish version has a Cronbach value of 0.81 (Muhonen and Torkelson, 2005). The 2-factorial structure of LOT-R, with one subscale for optimism and one for pessimism, has been shown to remain adequately stable across 2 years among Swedish women diagnosed with breast cancer (Saboonchi et al., 2016).

#### Coping Orientation to Problems Experienced, short version

We used a 28-item short version of the Coping Orientation to Problems Experienced (COPE) Inventory, known as the Brief-COPE, assessing an individual’s tendency to use different coping strategies when asked the main question, “What do you usually do when you are stressed by a problem?” (Carver, 1997). The instrument consists of 14 different scales, each representing a specific coping strategy: active coping, planning, positive reframing, acceptance, humor, religion, using emotional support, using instrumental support, self-distraction, denial, venting, substance use, behavioral disengagement, and self-blame.

We used the Swedish version of Brief-COPE, translated and validated by Muhonen and Torkelson (2005). The different coping strategies are presented through statements, and the respondents are asked to provide the most applicable answer. Each statement is graded on a 4-point Likert scale ranging from 1 (very seldom) to 4 (very often). The Swedish version of the Brief-COPE is psychometrically tested and shows adequate properties, with 12 out of the 14 scales having a Cronbach value between 0.58 and 0.92 (Muhonen and Torkelson, 2005). Acceptance and Denial scored lower, with 0.42 and 0.48, respectively.

#### Derriford Appearance Scale 24

We used the Derriford Appearance Scale 24 (DAS24) to measure adjustment among adults to problems related to visible differences, also called appearance-related distress (Carr et al., 2005). The DAS24 questionnaire was created to be used with populations of patients whose medical and surgical conditions may cause disfigurement or other appearance-related concerns (Carr et al., 2005).

The DAS24 consists of 24 items designed to measure distress and dysfunction in relation to appearance. The scale has a maximum score of 96, where a higher score indicates more distress (Carr et al., 2005). Cronbach value was 0.95, and test–retest reliability was good (0.82) (Carr et al., 2005). The DAS24 has not yet been validated in a Swedish population, but it has previously been used for populations with cleft, for example, in the United Kingdom and Australia (Roberts and Mathias, 2012; Moss et al., 2015). It has also been used in populations with other congenital malformations, as well as scarring from burns and trauma and in populations undergoing rhinoplasty (Carr et al., 2005; Günel and Omurlu, 2015). The result of DAS24 in the present population has been previously published by the first author (Paganini et al., 2020).

### Statistical Analyses

Statistical analyses were conducted using SAS version 9.4. For comparison between the 2 groups, men and women, Mann-Whitney *U* test was used for continuous variables and χ^2^ test was used for nonordered categorical variables. Spearman rank-order correlation coefficient was used to measure relationships between the scores of DAS24 and the scores of Brief-COPE and LOT-Rs.

The distribution of the scores of DAS24 was skewed, but when log (DAS24) was used, the residual variance was normally distributed. A multivariable general linear model, analysis of covariance (ANCOVA), was used to analyze the association between the independent possible contributing factors (the domains of Brief-COPE and LOT-Rs) and the dependent outcome variable (log (DAS24)). To find independent predictors to the outcome variable, interactions between gender and each of the other predictors were investigated one at a time, using the multivariable general linear model. All significance tests were 2 tailed and conducted at a 5% significance level. Effect sizes were calculated according to Cohen (1992) and were defined as low, medium, and large, that is, 0.20, 0.50, and 0.80, respectively (Cohen, 1992).

### Ethical Considerations

This study was approved by the regional ethical review board in Gothenburg (application number 970-11). The procedures followed were in accordance with the Helsinki Declaration of 1964, as revised. The participants gave their written informed consent to participate in the study.

## Results

After excluding one man who only responded to a portion of the study questionnaires, a total of 80 individuals were included in the study, 50 men and 30 women. The response rate was 50%. The mean age of the men and women was 38.8 and 37.4 years, respectively. The age distribution and available sociodemographic data are displayed in Table 1 (also reported in the study by Paganini et al., 2020).

**Table 1.** Demographic Data.

	Male (n = 50), n (%)	Female (n = 30), n (%)
Age, mean (SD)	38.8 (6.4)	37.4 (6.6)
Living arrangements major part of the week		
Living alone	16 (32.0)	3 (10.0)
Living with parents, siblings	1 (2.0)	1 (3.3)
Living with husband/wife or domestic partner	12 (24.0)	6 (20.0)
Living with children	0 (0)	4 (13.3)
Living with husband/wife or domestic partner and children	21 (42.0)	16 (53.3)
Level of education		
Did not finish 9-year compulsory school	1 (2.0)	0 (0.0)
Nine-year compulsory school	1 (2.0)	2 (6.9)
Senior high school	25 (50.0)	15 (51.7)
University exam	23 (46.0)	12 (41.4)
Occupation		
Working	41 (82.0)	20 (66.7)
Studying	1 (2.0)	2 (6.7)
On disability	1 (2.0)	1 (3.3)
Retired	0 (0.0)	2 (6.7)
On parental leave	0 (0.0)	2 (6.7)
Unemployed	2 (4.0)	1 (3.3)
Other	5 (10.0)	2 (6.7)

^a^ Previously published in the study by Paganini et al (2020).

When comparing the scoring of LOT-Rs between genders, a statistically significant difference was found in the domain “optimism,” where men scored higher than women, 5.90 (SD: 1.94) versus 4.94 (SD: 1.7), *P* = .037). The effect size was 0.50. In regard to the domains of Brief-COPE, one significant difference between genders was found in the domain “using emotional support,” where women scored higher than men, 5.90 (SD: 1.94) versus 4.94 (SD: 1.7), *P* = .018. The effect size was 0.54. No other significant gender differences were found in the mean scoring of Brief-COPE. See Table 2 for mean (SD) responses by gender of LOT-Rs and Brief-COPE.

**Table 2.** Mean Result (SD) of the DAS-24, LOT-Rs, and Brief-COPE by Domain and Gender.

	Males, mean (SD), n = 50	Females, mean (SD), n = 30	*P* value	Effect size
Derriford Appearance Scale 24^a^				
DAS24	28.8 (6.5)	40.2 (17.6)	<.001^b^	0.98
Domains of LOT-Rs^c^				
Optimism	8.55 (2.68)	7.10 (3.21)	.037^b^	0.50
Pessimism	3.87 (3.14)	4.83 (3.54)	.22	0.29
Domains of Brief-COPE^d^				
Active coping	6.10 (1.51)	6.50 (1.72)	.17	0.25
Planning	6.12 (1.58)	6.23 (1.68)	.79	0.07
Positive reframing	5.61 (1.44)	5.73 (1.82)	.56	0.08
Acceptance	6.06 (1.29)	6.13 (1.66)	.58	0.05
Humor	5.18 (1.89)	4.37 (1.83)	.05	0.43
Religion	2.78 (1.50)	2.93 (1.93)	.90	0.09
Using emotional support	4.94 (1.70)	5.90 (1.94)	.018^b^	0.54
Using instrumental support	4.59 (1.56)	5.23 (1.68)	.10	0.40
Self-distraction	4.18 (1.58)	4.63 (1.52)	.19	0.29
Denial	2.80 (0.94)	2.90 (1.30)	.89	0.09
Venting	4.57 (1.53)	4.97 (1.63)	.18	0.25
Substance use	2.39 (1.13)	2.30 (1.15)	.49	0.08
Behavioral disengagement	2.63 (1.17)	3.00 (1.31)	.18	0.31
Self-blame	4.55 (1.70)	4.87 (1.83)	.46	0.18

Abbreviations: Brief-COPE, 28-item short version of the Coping Orientation to Problems Experienced; DAS-24, Derriford Appearance Scale 24; LOT-Rs, revised Life Orientation Test, short version.

^a^ Scoring of DAS24 ranges from 11 to 96, wherein a higher score indicates higher levels of appearance-related distress.

^b^ Statistically significant group difference, *P* < .05.

^c^ Scoring of LOT-Rs ranges from 0 to 12, wherein a higher score indicates greater optimism/pessimism.

^d^ Scoring of Brief-COPE ranges from 2 to 8, wherein a higher score shows a higher inclination to use the indicated coping strategy.

Correlations between the scores of DAS24 and those of the domains of Brief-COPE and LOT-Rs were investigated. As shown in Table 3, the strongest correlations were found among females with the coping strategy “self-blame” (*r*_s_ =0.59, *P* < .001) and the domains “optimism” (*r*_s_ = −0.56, *P* = .001) and “pessimism” (*r*_s_ =0.59, *P* < .001) of the LOT-Rs. Among males, several coping strategies were found to be significant, encompassing the coping strategies “active coping” (*r*_s_ = −0.35, *P* = .014), “using emotional support” (*r*_s_ = −0.29, *P* = .044), “denial” (*r*_s_ = 0.39, *P* = .005) and “behavioral disengagement” (*r*_s_ = 0.41, *P* = .003), as well as the domain “pessimism” (*r*_s_ = 0.28, *P* = .046). Notable is that the correlations are weaker for men than for women.

**Table 3.** Correlation Between the DAS24 Score and the Choice of Coping Strategy and Level of Dispositional Optimism/Pessimism, Using Brief-COPE and LOT-Rs, Categorized by Gender.^a^

Domains	Derriford Appearance Scale-24 (DAS24)					
	Male (n = 50)		Female (n = 30)		Total (n = 80)	
	*r*_s_	*P* value	*r*_s_	*P* value	*r*_s_	*P* value
Brief-COPE						
Active coping	−0.35	.014^b^	0.07	.72	−0.14	.22
Planning	−0.14	.33	−0.10	.62	−0.11	.34
Positive reframing	−0.08	.58	−0.09	.64	−0.02	.86
Acceptance	−0.04	.77	0.03	.86	0.03	.82
Humor	0.01	.96	−0.19	.32	−0.11	.34
Religion	0.04	.78	−0.06	.77	−0.01	.92
Using emotional support	−0.29	.044^b^	−0.25	.19	−0.12	.31
Using instrumental support	−0.13	.36	−0.29	.12	−0.09	.43
Self-distraction	0.22	.12	0.22	.25	0.22	.05^b^
Denial	0.39	.005^b^	0.21	.27	0.29	.008^b^
Venting	0.24	.10	−0.21	.26	0.14	.21
Substance abuse	0.05	.72	0.34	.07	0.10	.39
Behavioral disengagement	0.41	.003^b^	0.36	.05	0.39	<.001^b^
Self-blame	0.27	.06	0.59	<.001^b^	0.41	<.001^b^
LOT-Rs						
Optimism	−0.24	.09	−0.56	.001^b^	−0.42	<.001^b^
Pessimism	0.28	.046^b^	0.59	<.001^b^	0.43	<.001^b^

Abbreviations: Brief-COPE, 28-item short version of the Coping Orientation to Problems Experienced; DAS-24, Derriford Appearance Scale 24; LOT-Rs, revised Life Orientation Test, short version.

^a^ Spearman rank-order correlation coefficient is used.

^b^ Statistically significant group difference, *P* < .05.

Since the correlation analysis showed that some domains had a greater impact on appearance-related distress, a multivariable linear regression analysis was conducted to explore how the correlated domains affected DAS24 scores. The domains found to be significantly correlated in Table 4 were included in the multivariable linear regression, and the log score of DAS24 was entered as the dependent variable.

**Table 4.** Multiple Regression Analyses.^a,b,c^

	Parameter estimate (SE)	*P* value
Intercept	2.31 (0.30)	<.001^d^
Gender	0.60 (0.15)	<.001^d^
Brief-COPE self-distraction	0.016 (0.017)	.36
Brief-COPE denial	0.071 (0.027)	.01^d^
Brief-COPE behavioral disengagement	0.005 (0.026)	.84
Brief-COPE self-blame	0.033 (0.017)	.053
LOT-Rs pessimism	0.010 (0.010)	.34
LOT-Rs optimism	0.047 (0.030)	.12
LOT-Rs optimism × gender	−0.051 (0.018)	.007^b^

Abbreviations: Brief-COPE, 28-item short version of the Coping Orientation to Problems Experienced; DAS-24, Derriford Appearance Scale 24; LOT-Rs, revised Life Orientation Test, short version; SE, standard error.

^a^ Dependent variable: log(DAS24). Interaction term: LOT-Rs optimism × gender.

^b^ *R* ^2^ = 0.55, Durbin Watson *D* = 2.23.

^c^ The interaction effect gender is coded: 1 = male and 2 = female, making the β-coefficient for males 0.047 − 0.051 = −0.004 and the β-coefficient for females 0.047 + 2 (−0.051) = −0.056.

^d^ Statistically significant group difference, *P* < .05.

The regression analysis shows that 55% of the variance of log (DAS24) can be explained by gender, Brief-COPE self-distraction, Brief-COPE denial, Brief-COPE behavioral disengagement, Brief-COPE self-blame, LOT-Rs pessimism, LOT-Rs optimism, and the interaction variable LOT-Rs optimism × gender, as seen in Table 4. Revised Life Orientation Test, short version optimism was the only predictor with significant interaction with gender.

The interaction variable, optimism × gender, in the ANCOVA shows that LOT-Rs (optimism) has a gendered effect on log (DAS24), wherein the β coefficient for males and females was –0.004 and –0.056, respectively, when gender was coded as 1 = male and 2 = female (Table 4). Thus, appearance-related distress is more affected by dispositional optimism among women than men.

## Discussion

The present study aimed to investigate whether gender differences in appearance-related distress among individuals with UCLP can be understood through levels of dispositional optimism as well as choice of coping strategies. The questionnaires LOT-Rs and Brief-COPE were used to describe the levels of dispositional optimism and coping strategies in a population of adults born between 1966 and 1986 with UCLP. Appearance-related distress among individuals with UCLP, as measured by the DAS24, differed between genders. Women reported significantly more distress than men in this sample, which concurs with previous studies of populations with visible differences (Rumsey et al., 2004; Carr et al., 2005; Roberts and Mathias, 2012) and can also be seen within the general population (Wang et al., 2019; Lacroix et al., 2020).

In the present study, a significant difference was found in the scores of dispositional optimism in the LOT-Rs between men and women, wherein men were more optimistic than women. This differs from population-based studies of noncleft individuals, where female participants generally tend to be more positive than male participants (Gallagher et al., 2013; Hinz et al., 2017; Schou-Bredal et al., 2017). It is notable that the women in this sample were less optimistic and more pessimistic than the norm population, while the differences were not as prominent for the men. The lower levels of optimism among women could indicate a lower degree of psychosocial well-being (Carver and Scheier, 2014; Scheier and Carver, 2018). Since the women in the sample were also found to be more dissatisfied with their appearance, the influence of lower optimism on appearance-related distress can be assumed to be of importance. A previous study using LOT-Rs among parents of children born with cleft showed that a positive life orientation lowers the impact of the cleft on the family (Stock et al., 2020c), which could possibly be extrapolated to the adult population with cleft, but further studies are needed to confirm this.

In terms of the coping strategies measured using Brief-COPE, a significant difference was found in the coping domain, “using emotional support,” wherein women scored significantly higher than men. Otherwise, the differences were small and not statistically significant. This is consistent with prior literature, according to which gender differences regarding choice of coping strategy are generally small, and the most robust difference found is that women are more likely than men to seek emotional support (Tamres et al., 2002). It has previously been shown that women report a greater use of and a greater variety of coping strategies than men (Tamres et al., 2002). In the present study, the same pattern was found, where women reported higher utilization rate than men in all coping scales, except for “humor” and “substance use.”

To further investigate the relationship between appearance-related distress and dispositional optimism as well as coping strategies, a correlation analysis was undertaken. The correlation analysis revealed that some domains of the LOT-Rs and Brief-COPE have greater influence on the scores of DAS24 than others, and differences between genders were found. Among the women in the study, the strongest correlations were found with the coping strategy “self-blame” together with the domains “optimism” and “pessimism.” The correlations were rather robust and showed that, among women, a higher amount of appearance-related distress is correlated with lower levels of optimism. Among men, several coping strategies were found to be significant, including “behavioral disengagement,” “denial,” and the domain “pessimism.” However, the correlations with appearance-related distress were weaker among men than women, suggesting that high appearance concerns among men were not as strongly associated with their coping strategies and dispositional optimism as for women.

To further explore how the correlated items affect the scores of DAS24, a multivariable linear regression analysis was carried out. The results showed that 55% of the variance in appearance-related distress was explained by “gender,” “optimism,” “denial,” and “self-blame” and the interaction of “gender × optimism.” The only significant gender difference observed was in regard to LOT-Rs, wherein the optimism among women affected the scores of DAS24 to a higher degree than men. Therefore, a positive change in optimism had a greater effect on mitigating appearance-related distress among women compared to men in this sample.

The clinical implications of this study are that clinicians meeting individuals born with cleft need to be aware that personality traits and coping strategies play a role in appearance-related distress, especially during late adolescence and early adulthood. It is difficult to change a personality trait in a person, such as dispositional optimism, and without intervention or major life transitions, traits stay relatively stable over the lifespan. Methods using cognitive behavioral therapy seem to raise levels of optimism at least temporarily (Carver and Scheier, 2014), but it is difficult to obtain lasting results. However, it can be possible to strengthen the more dynamic parts of an individual, such as coping strategies like mindfulness-based techniques which help in coping with both everyday distress and more extraordinary situations (Grossman et al., 2004; Abbott et al., 2014). The present study showed that coping differed between genders and that the use of coping strategies was correlated with appearance-related distress. This creates the opportunity to tailor gender specific guidance, with a focus on minimizing the use of “self-blame” as a coping strategy for appearance for women, while men can be helped to minimize the feeling of “denial” as well as “behavioral disengagement”. The cleft team can, during the treatment period, incorporate ways to increase psychosocial competence in identifying and supporting positive coping skills, such as mindfulness or cognitive-based techniques. The cleft team has a rare advantage in that it follows families and children from infancy to adulthood, allowing for ample opportunity to provide interventions and support over a long period of time.

One limitation of this study is the response rate of 50%, which, although low, is acceptable. Other studies using questionnaires, including samples of individuals with UCLP, have reported similar or lower response rates (Sinko et al., 2005; Nicholls et al., 2018). In addition, women were more likely to participate in the study than men (51% vs 42%), even though the number of eligible men was higher due to the gender difference in the prevalence of UCLP. Regarding the participants’ experience with UCLP, it was unknown what treatment was provided outside the standardized CLP surgeries. A major constraint was the lack of a matched control group of individuals without UCLP, which would have increased the ability to interpret the results beyond comparison to norm groups. The need of a control group, as well as validation in a Swedish population, is highlighted when considering the large difference in the scores of DAS24 between men and women. This difference is not as prominent in the British norm-material (Carr et al., 2005), which emphasizes the need for Swedish norms. Additionally, the Swedish version of Brief-COPE included 2 scales with low reliability, “acceptance” and “denial,” and the Swedish Brief-COPE needs to be studied further. This study also reveals the need for further research on how to deliver interventions that account for gender and improve an individual’s optimism and coping strategies.

## Conclusion

In conclusion, the main finding of this study indicates that dispositional optimism influenced appearance-related distress, wherein a higher level of optimism decreased the experienced distress. This tendency was stronger among women, as appearance-related distress among men was not affected by optimism to the same degree. It was also shown that appearance-related distress was correlated with the use of coping strategies, which differed by gender. Appearance-related distress among women was affected by the use of “self-blame,” whereas among men, correlations were found in regard to “active coping,” “using emotional support,” “denial,” and “behavioral disengagement.”

These findings may have clinical implications in terms of helping clinicians to better understand and help their patients manage or reduce their potential psychosocial distress, as well as understanding how gender influences distress. It is important to ensure that the cleft team includes the provision of psychosocial care, for both screening of distress and promoting optimism and adaptive coping strategies.
